# Malnutrition-sarcopenia syndrome predicts mortality in hospitalized older patients

**DOI:** 10.1038/s41598-017-03388-3

**Published:** 2017-06-09

**Authors:** Xiaoyi Hu, Lei Zhang, Haozhong Wang, Qiukui Hao, Birong Dong, Ming Yang

**Affiliations:** 1 The Center of Gerontology and Geriatrics, West China Hospital, Sichuan University, No. 37 Guoxue Lane, Chengdu, Sichuan China; 2 The Department of Orthopedic Surgery, West China Hospital, Sichuan University, No. 37 Guoxue Lane, Chengdu, Sichuan China

## Abstract

A new term, malnutrition-sarcopenia syndrome (MSS), was recently coined to describe the clinical presentation of both malnutrition and sarcopenia. The aim of this study was to investigate the association between MSS and long-term mortality in older inpatients. We conducted a prospective study in acute geriatric wards of two local hospitals in China. Muscle mass and malnutrition were estimated by anthropometric measures and the Mini Nutritional Assessment (MNA). Of the 453 participants, 14 (3.1%) had sarcopenia with normal nutrition, 139 (30.7%) had malnutrition risk without sarcopenia, 48 (10.6%) had malnutrition risk with sarcopenia, 25 (5.5%) had malnutrition without sarcopenia, and 22 (4.9%) had MSS at baseline. Compared with non-sarcopenic subjects with normal nutrition, subjects with MSS and subjects with malnutrition risk and sarcopenia were more than four times more likely to die (hazard ratio [HR], 4.78; 95% confidence interval [CI], 2.09–10.97; and HR, 4.25; 95% CI, 2.22–8.12, respectively); non-sarcopenic subjects with malnutrition risk were more than two times more likely to die (HR, 2.41; 95% CI, 1.32–4.39). In conclusion, MSS may serve as a prognostic factor in the management of hospitalized older patients.

## Introduction

Malnutrition is common in different populations, but especially in older adults^1^. Older people with malnutrition (or malnutrition risk) are at increased risk of adverse clinical outcomes, such as prolonged length of hospital stay, increased health costs, poor quality of life, and mortality^2^.

Sarcopenia is a new geriatric syndrome that comprises age-related loss of muscle mass, strength, and performance^3^. Sarcopenia is also prevalent in older adults, especially among those in health care settings^4^. Furthermore, sarcopenia is also associated with many adverse health outcomes, including death^5^. Although malnutrition and sarcopenia may share some clinical features, such as abnormal body weight or inactivity^6^, the fundamental mechanisms of the two conditions differ^7^.

Based on these facts, Vandewoude and colleagues recently suggested using a new term, “malnutrition-sarcopenia syndrome (MSS)”, to describe the clinical presentation of both malnutrition and sarcopenia^7^. This concept has not yet been widely accepted, and its value for clinical practices and research remains unclear. In hospitalized older patients, both malnutrition^8^ and sarcopenia^9, 10^ are highly prevalent and have been proven to increase the risk of death; therefore, we speculated that older inpatients with both syndromes (i.e., MSS) were at higher risk of mortality than those only suffering from either malnutrition or sarcopenia. We conducted a prospective study with the following objectives: 1) to identify the prevalence of malnutrition, sarcopenia, and MSS in a study population of hospitalized older patients; and 2) to investigate the association between MSS and long-term mortality in this population.

## Methods

We conducted a prospective study in the acute geriatric wards of two local hospitals located in Chengdu, China: West China Hospital of Sichuan University and the Fifth People’s Hospital of Chengdu City. The study protocol was approved by the Research Ethics Committee of Sichuan University. A written informed consent was obtained from all participants or their legal proxies. All methods in this study were in accordance with relevant regulations and guidelines.

### Baseline study population

During August to December 2012, we recruited the consecutively admitted older patients (aged 60 years or older) from the acute geriatric wards of the two hospitals to participate in this study. Patients with the following conditions at admission were excluded: 1) end-of-life diseases; 2) clinically visible edema; 3) severe cognitive impairment, and 4) delirium.

### Data collection

Trained interviewers performed face-to-face interviews to obtain the baseline data from all participants within 48 hours after admission. In addition, the following anthropometric measurements were performed by trained technicians: body weight, height, mid-arm circumference (MAC), and calf circumference (CC).

### Sarcopenia assessment

**Figure 1:**
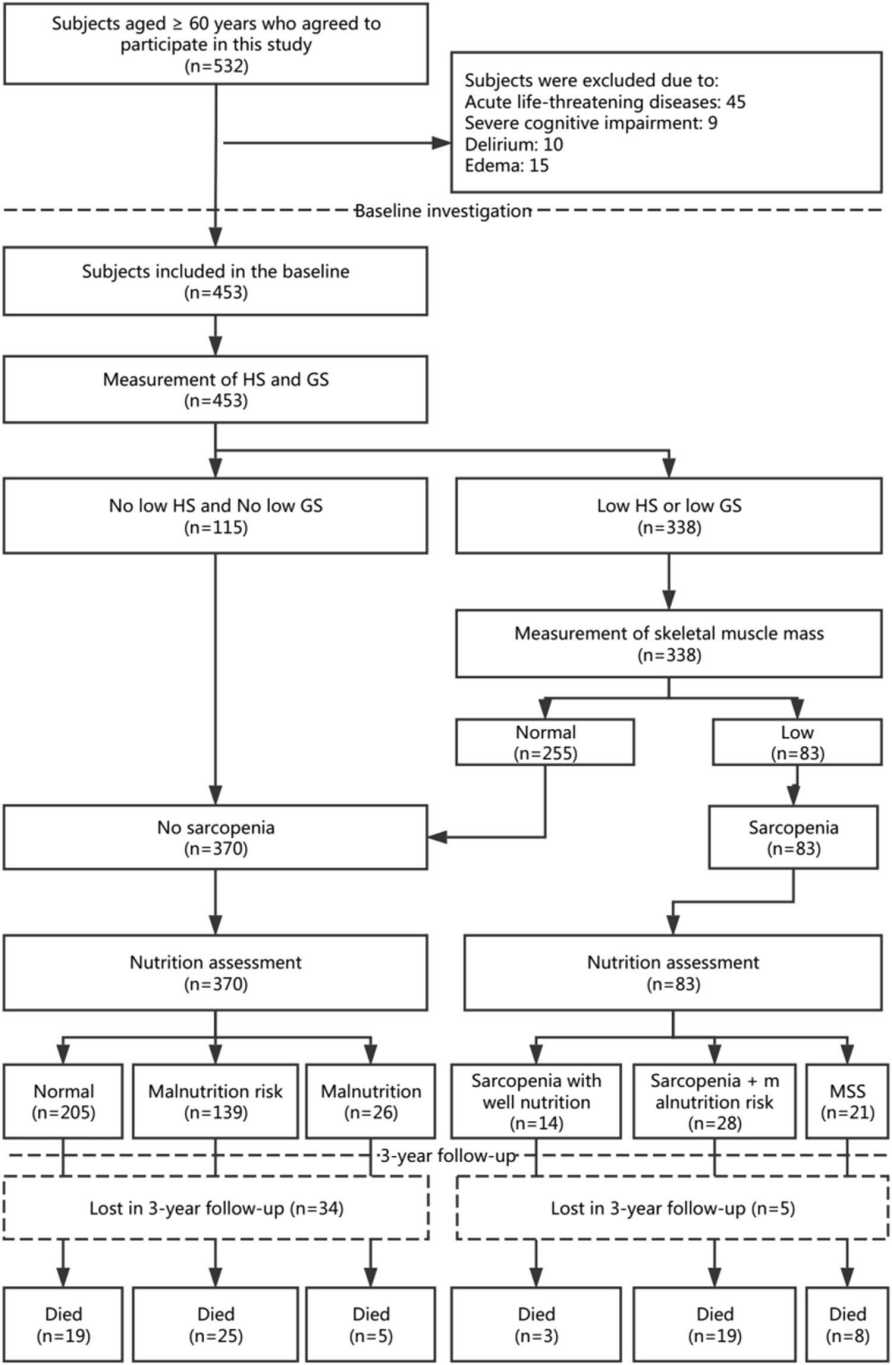
Study profile using the diagnostic algorithm from the Asian Working Group for Sarcopenia (AWGS).

The recommended diagnostic algorithm of the Asia Working Group for Sarcopenia (AWGS)^11^ was applied to define sarcopenia. According to the AWGS recommendation, patients with low muscle mass combined with low handgrip strength (HS) and/or a low gait speed (GS) were considered to have sarcopenia (Fig. 1).

### Muscle mass estimation

The appendicular skeletal muscle mass (ASM) was estimated using an equation previously validated in a Chinese population^12^:

$${\rm{ASM}}=0.193\times {\rm{body}}\,{\rm{weight}}+0.107\times {\rm{height}}-4.157\times {\rm{gender}}-0.037\times {\rm{age}}-\mathrm{2.631.}$$

The body weight, height, and age were measured in kilograms, centimeters, and years, respectively. For gender, a value of 1 represented men, and a value of 2 represented women^12^. The agreement of the ASM equation model and dual X-ray absorptiometry (DXA) was good (adjusted R^2^ = 0.90, standard error of estimate (SEE) = 1.63 kg)^12^.

Thereafter, the muscle mass index (SMI) was calculated using the following equation: SMI = ASM/height^2^. Similar to previous studies^13–15^, the cut-off for defining low muscle mass was based on the SMI of the lowest 20th% percentile of the study population. Therefore, a low muscle mass was defined as an SMI less than 6.92 kg/m^2^ in men and less than 5.13 kg/m^2^ in women.

### Handgrip strength measurement

The HS was measured by trained technicians using a handheld dynamometer based on strain gauge sensors (EH101, Xiangshan Inc., Guangdong, China) to the nearest 0.1 kg. According to the AWGS consensus, a low HS was defined as less than 26 kg for men and less than 18 kg for women.

### Gait speed measurement

As recommended by the AWGS^11^, a 4-meter walking test was performed to measure the usual gait speed. Canes or walkers were accepted during the walking test, if necessary. A low GS was defined as less than 0.8 m/s^11^.

### Nutritional status assessment

We used the Mini Nutritional Assessment (MNA)^16^ to assess the participants’ nutritional status. The MNA is “the most widely used tool for nutritional screening and assessment of the elderly”^16^. It has been validated in different populations including Chinese^17^. The total score of the MNA is 30 points, a higher score is preferable. Subjects with scores of 23.5 or more were classified as normally nourished; a score between 17 and 23.5 indicated that a subject had “malnutrition risk”; and a score of less than 17 indicated that a subject had malnutrition^16^.

It is noteworthy that the BMI is included in both the MNA and the aforementioned SMI equation as a component. However, the MNA includes 18 items with a total of 30 points and the BMI is only one item of the MNA (with a maximum of 3 points). Therefore, we hypothesized that nutrition status, which is defined by the MNA, would not be significantly influenced by the BMI. To support our hypothesis, we also defined the nutrition status by using the modified “MNA without BMI” that has been validated previously in Taiwanese^18, 19^. And we compared the nutrition status defined by the MNA and the modified “MNA without BMI”.

### Identification of other confounders

We collected the following covariates from the face-to-face interviews: age, gender, education level, physical activity ≥30 min/d, smoking status, and alcohol drinking status. We also identified the following conditions using the hospital information systems: hypertension, ischemic heart disease, chronic obstructive pulmonary disease (COPD), diabetes, stroke, chronic kidney disease, acute infection, tumor of any type, osteoarthritis, liver disease, gastrointestinal disease, urinary incontinence, chronic pain, and receiving nutritional supplements during hospitalization. Furthermore, we evaluated depression and cognitive function using the Chinese version of the 30-item Geriatric Depression Scale (GDS-30)^20^ and the Chinese version of the Mini-Mental Status Examination (MMSE)^21^, respectively. The Older Americans Resources and Services (OARS) multidimensional functional assessment questionnaire^22^ was applied to assess the activity of daily living (ADL) and instrumental activity of daily living (IADL) of each participant.

### Follow-up

The survival status of the participants was collected by telephone interviews at 12, 24, and 36 months during the 3-year follow-up period. These survival data were also confirmed using the Local Death Registry Database. Time to death was calculated as the time between the first interview and the date of death.

### Statistical analyses

We presented the categorical data as absolute number and percentages (%) and the continuous data as the mean ± standard deviation (SD). We applied the Pearson chi-squared test for categorical data and the one-way ANOVA for continuous data to compare the differences between groups. A p value less than 0.05 was considered statistically significant. We applied Cox regression models with a backward stepwise selection to calculate the adjusted hazard ratios (HR) and 95% confidence intervals (CI) and identify independent prognostic factors for all-cause mortality. The initial model of backward stepwise selection included the following covariates: age, gender, education level, smoking status, alcohol drinking status, physical activity status, conditions listed above, ADL scores, IADL scores, GDS-30 scores, and MMSE scores. In these models, the criteria for entry and removal were p < 0.05 and p > 0.10, respectively. Multiplicative interaction was evaluated by creating a cross-product of each aforementioned covariate and the “nutrition combined with sarcopenia” status. And we included these cross-product terms, along with their main effects and the other covariates, in a Cox proportional hazard model. In addition, Survival curves were estimated using the Kaplan–Meier method. Survival curves were compared using log-rank tests. Statistical analysis was performed using SPSS 20.0 (IBM SPSS Statistics, Armonk, NY, USA).

## Results

### Description of the study population

A total of 453 subjects were included in the baseline analyses (Fig. 1). In the baseline investigation, the mean age of the study population was 79.0 ± 7.8 years (range from 60 to 101 years), and 135 subjects (29.8%) were women. All participants were Han Chinese. During the 3-year follow-up, 39 subjects (8.6%) were lost to follow-up, which led to a final sample of 414 subjects. The participants lost to follow-up were balanced between the sarcopenia group and the non-sarcopenia group (9.2% versus 6.0%, p = 0.353).

### Characteristics of participants with or without sarcopenia

Based on the recommendation of the AWGS, 83 participants (18.3%) were found to have sarcopenia at the baseline investigation. The prevalence of sarcopenia was similar between men and women (18.9% versus 17.0%, respectively; p = 0.645). Supplementary Table S1 presents the characteristics of participants according to sarcopenia status.

### Characteristics of participants with different nutrition status

Based on the results of the MNA scale, the prevalence of malnutrition risk and malnutrition were 41.3% and 10.4%, respectively. Men were more prone to suffered from malnutrition risk than women (44.7% versus 33.3%, p = 0.033), however, there was no significant difference between men and women with respect to malnutrition (9.4% versus 12.6%, p = 0.076). Table 1 presents the characteristics of participants according to their nutritional status.

**Table 1:** Baseline characteristics of participants according to nutrition status.

Characteristic	Normal nutrition (n = 219)	Malnutrition risk (n = 187)	Malnutrition (n = 47)	p
Age (years)	78.7 ± 8.0	79.4 ± 8.0	79.3 ± 6.3	0.677
Women	73 (33.3)	45 (24.1)	17 (36.2)	0.076
Education level				
Unschooled	16 (7.3)	19 (10.2)	8 (17.0)	0.060
Primary school	50 (22.8)	41 (21.9)	16 (34.0)	
High school or above	153 (69.9)	127 (67.9)	23 (48.9)	
Current smokers	23 (10.5)	25 (13.4)	6 (12.8)	0.683
Current alcohol drinkers	28 (12.8)	22 (11.8)	4 (8.5)	0.154
Physical activity ≥30 min/d	146 (66.7)	91 (48.9)	19 (40.4)	<0.001
Comorbidities				
Hypertension	137 (62.6)	94 (50.3)	18 (38.3)	0.002
Ischemic heart disease	68 (31.1)	54 (28.9)	10 (21.3)	0.406
COPD	55 (25.1)	64 (34.2)	19 (40.4)	0.041
Diabetes	55 (25.1)	53 (28.3)	10 (21.3)	0.558
Stroke	10 (4.6)	16 (8.6)	2 (4.3)	0.212
CKD	29 (13.2)	24 (12.8)	4 (8.5)	0.668
Acute infection	59 (26.9)	52 (27.8)	14 (29.8)	0.921
Osteoarthritis	51 (23.3)	48 (25.7)	13 (27.7)	0.760
Tumor of any type	16 (7.3)	27 (14.4)	5 (10.6)	0.067
GI disease	42 (19.2)	35 (18.7)	12 (25.5)	0.559
Liver disease	17 (7.8)	15 (8.0)	4 (8.5)	0.984
Urinary incontinence	17 (7.8)	27 (14.4)	6 (12.8)	0.094
Chronic pain	59 (26.9)	52 (27.8)	14 (29.8)	0.921
Nutritional supplements	2 (0.9)	6 (3.2)	40 (85.1)	<0.001
Sarcopenia	14 (6.4)	48 (25.7)	21 (44.7)	<0.001
BMI (kg/m^2^)				
Women	24.2 ± 3.5	21.2 ± 4.2	18.6 ± 2.4	<0.001
Men	24.3 ± 3.2	20.9 ± 3.1	19.5 ± 3.6	<0.001
CC (cm)				
Women	33.1 ± 4.3	30.8 ± 4.2	29.1 ± 3.3	0.001
Men	34.3 ± 3.7	31.6 ± 3.5	31.1 ± 4.1	<0.001
MAC (cm)				
Women	27.4 ± 3.8	25.8 ± 3.9	24.8 ± 3.3	0.026
Men	27.1 ± 3.5	25.8 ± 3.2	25.4 ± 3.7	0.003
Gait speed (m/s)				
Women	0.7 ± 0.3	0.7 ± 0.3	0.6 ± 0.2	0.522
Men	0.8 ± 0.4	0.8 ± 0.3	0.7 ± 0.2	0.621
Handgrip strength (kg)				
Women	15.8 ± 6.9	12.8 ± 6.9	10.8 ± 7.0	0.033
Men	23.7 ± 7.0	21.9 ± 8.3	20.6 ± 13.9	0.013
SMI (kg/m^2^)				
Women	5.8 ± 0.7	5.2 ± 0.8	4.7 ± 0.6	<0.001
Men	7.6 ± 0.6	6.9 ± 0.6	6.7 ± 0.7	<0.001
ADL scores	7.7 ± 2.3	9.6 ± 4.0	10.8 ± 4.8	<0.001
IADL scores	9.1 ± 3.5	11.7 ± 5.2	12.9 ± 5.7	<0.001
GDS-30 scores	6.2 ± 4.6	8.2 ± 5.4	10.9 ± 5.8	<0.001
MMSE scores	26.1 ± 4.0	23.4 ± 6.1	21.0 ± 7.0	<0.001

Data are presented as the number (percent) for the following variables: women, education level, marital status, current smokers, current alcohol drinkers, physical activity, specific comorbidities, and sarcopenia. For other variables, the mean ± SD are used.

One-way ANOVA was used for continuous variables, and the Pearson chi-squared test was used for categorical variables. During testing, p < 0.05 was considered statistically significant.

ADL: activities of daily living; BMI: body mass index; CC: calf circumference; CKD: chronic kidney disease; COPD: chronic obstructive pulmonary disease; GDS-30: 30-item Geriatric Depression Scale; GI: gastrointestinal; IADL: instrumental activities of daily living; MAC: mid-arm circumference; MMSE: Mini-Mental Status Examination; SMI: skeletal muscle index.

When using the modified “MNA without BMI” scale, the prevalences of malnutrition risk and malnutrition were very similar (Supplementary Table S2).

### Association between sarcopenia and malnutrition

Compared with non-sarcopenic subjects, sarcopenic subjects were more prone to have malnutrition risk (57.8% versus 37.7%, p < 0.001) and malnutrition (25.3% versus 6.3%, p < 0.001). In addition, both sarcopenic men and women exhibited a lower BMI, MAC, and CC compared with their counterparts without sarcopenia (Supplementary Table S1). On the other hand, compared to subjects with normal nutrition, the prevalence of sarcopenia was significantly higher in subjects with malnutrition risk and in subjects with malnutrition (6.4%, 25.7%, and 44.7%, respectively, p < 0.001, Table 1).

**Figure 2:**
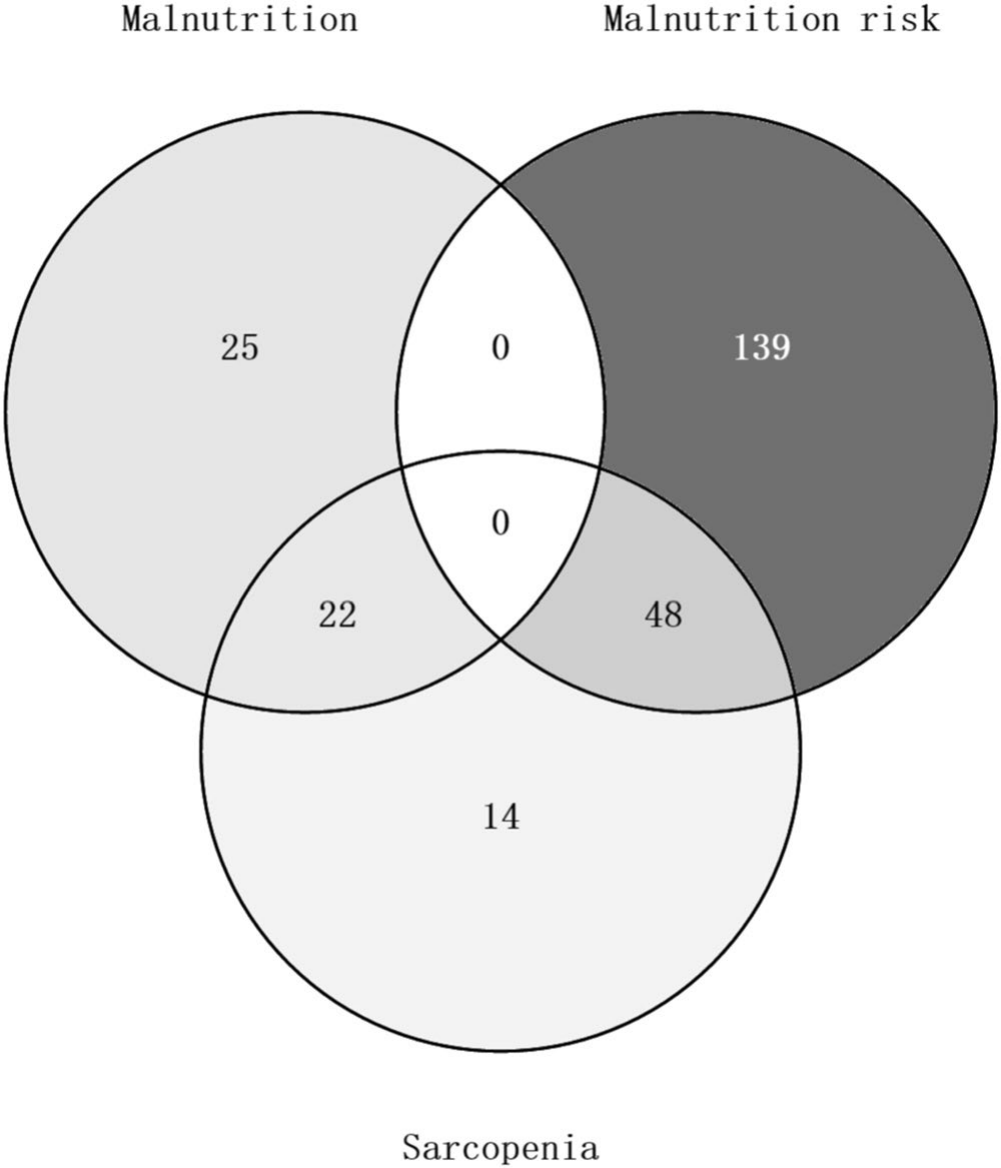
Venn diagram illustrating the overlap between malnutrition risk, malnutrition, and sarcopenia.

When considering the two syndromes together, 14 participants (3.1%) had sarcopenia with normal nutrition, 139 (30.7%) had malnutrition risk without sarcopenia, 48 (10.6%) had malnutrition risk and sarcopenia, 25 (5.5%) had malnutrition without sarcopenia, and 22 (4.9%) had MSS. Figure 2 displays the overlap between malnutrition risk (or malnutrition) and sarcopenia.

### Association between sarcopenia, malnutrition, and MSS with 3-year mortality

During the 3-year follow-up, 79 participants (19.1%) died (men: 21.8%, women: 12.5%, p = 0.029). The 3-year all-cause mortality was 9.3% in subjects with non-sarcopenia and normal nutritional status, 21.4% in sarcopenic subjects with normal nutritional status, 22.7% in non-sarcopenic subjects with malnutrition risk, 23.8% in non-sarcopenic subjects with malnutrition, 43.2% in sarcopenic subjects with malnutrition risk, and 38.1% in subjects with MSS (p < 0.001). Table 2 shows the model derived from the Cox regression analysis using a backward stepwise selection. Compared with normally-nourished subjects without sarcopenia, subjects with MSS were more than four times more likely to die (HR, 4.78; 95% CI, 2.09–10.97); subjects with both malnutrition risk and sarcopenia were also more than four times more likely to die (HR, 4.25; 95% CI, 2.22–8.12); non-sarcopenic subjects with malnutrition risk were more than two times more likely to die (HR, 2.41; 95% CI, 1.32–4.39); non-sarcopenic subjects with malnutrition were also more than two times more likely to die, but the measure of effect was not statistically significant (HR, 2.62; 95% CI 0.98–7.04); and sarcopenic subjects with normal nutritional status had no significantly higher risk of death (HR, 1.66; 95% CI, 0.48–5.72). When using the “modified MNA without BMI” scale to define malnutrition risk and malnutrition, the results were very similar (Supplementary Table S3).

**Table 2:** Factors associated with 3-year mortality according to the Cox Regression Model with a backward stepwise selection.

	Coefficient	SE	Wald	p	HR	95% CI for HR	
						Lower	Upper
Sarcopenia with normal nutrition	0.509	0.630	0.652	0.419	1.66	0.48	5.72
Malnutrition risk without sarcopenia	0.878	0.307	8.181	0.004	2.41	1.32	4.39
Malnutrition risk and sarcopenia	1.446	0.330	19.186	0.000	4.25	2.22	8.12
Malnutrition without sarcopenia	0.964	0.504	3.658	0.056	2.62	0.98	7.04
MSS	1.565	0.423	13.655	0.000	4.78	2.09	10.97
Age	0.054	0.018	8.943	0.003	1.06	1.02	1.09
Tumor of any type	0.650	0.292	4.953	0.026	1.92	1.08	3.39

CI: confidence interval; HR: hazards ratio; MSS: malnutrition-sarcopenia syndrome; SE: standard error.

In addition, the sensitivity analyses did not find any evidence of multiplicative interaction between each covariate and the “nutrition combined with sarcopenia” status on all-cause mortality (Supplementary Table S4).

**Figure 3:**
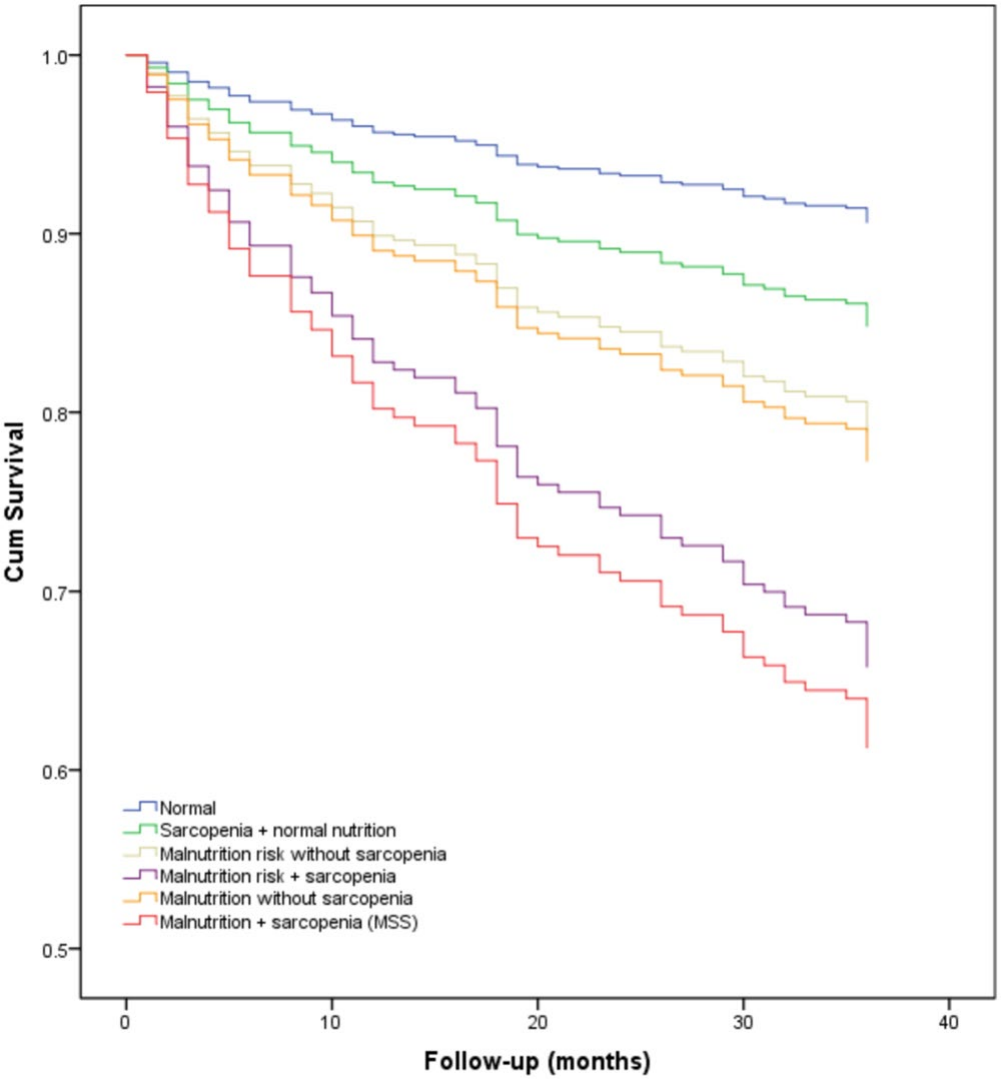
Survival curves of the study population according to sarcopenia and nutritional status at baseline. Survival curves significantly differed in the log-rank test (p < 0.001).

The survival curves of the subjects categorized by sarcopenia and nutritional status are presented in Fig. 3. These survival curves were significantly different by the log-rank test (p < 0.001). These survival curves indicated that subjects with MSS and subjects with both malnutrition risk and sarcopenia had increased risk of death during the 3-year follow-up compared with those only having either of the two syndromes. In addition, non-sarcopenic subjects with malnutrition or at risk of malnutrition had increased risk of death compared with sarcopenic subjects with normal nutritional status.

## Discussion

To the best of our knowledge, this is the first prospective study to address the value of MSS for the prediction of long-term all-cause mortality in hospitalized older patients. Our study demonstrated that malnutrition/ malnutrition risk and sarcopenia were prevalent in the study population of older inpatients, and there was an overlap between these two syndromes. After adjusting for clinical confounders, participants with malnutrition/ malnutrition risk or participants with sarcopenia appeared to have increased risk of 3-year all-cause mortality; however, participants with both of the two syndromes (MMS or malnutrition risk plus sarcopenia) were at even higher risk of 3-year all-cause mortality.

Malnutrition currently lacks clear and well-accepted diagnostic criteria^1^. Many screening tools have been developed for identifying malnutrition in older adults, such as the Nutritional Risk Screening 2002 (NRS-2002), the Malnutrition Universal Screening Tool (MUST), Subjective Global Assessment (SGA), and MNA^1^. According to a recent systematic review^22^, MNA performed fair to good in older people; MUST performed fair to good in adults; whereas SGA, NRS-2002 and MUST did not perform well in predicting health care outcomes in older patients. Therefore, we chose MNA for screening malnutrition in this study.

The association between malnutrition and mortality has been well documented in previous studies^7^. However, when focusing on malnutrition as defined by the MNA, inconsistent results were identified in previous studies. For example, a prospective study demonstrated that malnourished inpatients (assessed by the MNA) in acute geriatric wards had a higher risk of 1-, 2- and 3-year mortality compared with their counterparts with normal nutrition^23^. Similarly, another prospective study found that malnutrition defined by the MNA was an independent prognostic factor for long-term mortality in hospitalized geriatric patients with heart failure^24^. Our study found similar results in a study population of Chinese older inpatients. However, several other studies have found opposite results^25, 26^. For example, Vischer *et al*. reported that malnutrition, as defined by the MNA, failed to predict long-term mortality in older inpatients with a heavy disease burden^25^.

Well-accepted diagnostic criteria for sarcopenia is lacking. There are currently at least five international consensuses available for defining sarcopenia^27^. Because all participants were Han Chinese, we applied the diagnostic algorithm from the AWGS^11^ to define sarcopenia in this study. It is notable that the AWGS^11^ recommended using bioelectrical impedance analysis (BIA) or DXA to estimate muscle mass; however, we estimated muscle mass using a previously validated anthropometric equation in this study. The reason for this inconsistency is that BIA has recently been proven to overestimate muscle mass compared with DXA in older inpatients. Furthermore, the cut-off points for defining low muscle mass in Chinese older adults using BIA have not yet been established^28, 29^. On the other hand, DXA is expensive and associated with a risk of X-ray exposure. Furthermore, Alexandre *et al*. recently reported that the use of anthropometric equations to estimate muscle mass combined with the handgrip and gait speed constituted an important alternative to DXA for diagnosis of sarcopenia and reduced costs^15^.

This study indicated that older inpatients with MSS or with malnutrition risk and sarcopenia were at higher risk of long-term mortality when compared with those having either of the two syndromes alone; whereas sarcopenic subjects with normal nutrition had no significantly increased risk of death. These findings imply that it is valuable to assess sarcopenia together with nutritional status in the management of geriatric inpatients. Although there is no published study addressing the association between MSS and mortality, several studies did evaluate malnutrition and sarcopenia at the same time in various study populations. For example, a recent prospective study demonstrated that malnutrition and sarcopenia were separately and significantly associated with post-liver transplant morbidity and 1-year mortality^30^. Cerri *et al*. reported that sarcopenia was associated with short-term mortality in older inpatients with or at-risk for malnutrition^31^.

This study demonstrated that subjects with MSS and subjects with malnutrition risk and sarcopenia were at similarly increased risk of death. Similarly, non-sarcopenia subjects with malnutrition and with malnutrition risk were also at similar risk of death. These findings suggest that the identification of malnutrition risk in hospitalized older patients is at least as importance as the screening of malnutrition in either sarcopenic or non-sarcopenic individuals. Our study provides a confirmative answer to the question of whether subjects with malnutrition risk and sarcopenia fall within the MSS.

Several limitations need to be considered when interpreting the results of this study. First, although the diagnostic criteria of MSS remain unclear, Vandewoude and colleagues did offer an assessment tool for MSS based on their experience^7^. However, the reliability and validity of the assessment tool are unknown. In this study, we did not apply that assessment tool because we did not estimate the muscle mass by DXA, BIA, CT or MRI, as Vandewoude *et al*. required. Second, because of the study design, survival bias prior to entry in the cohort should be considered. Last, the sample size of the study population was relatively small, especially for those with MSS.

## Conclusion

In a population of geriatric inpatients, subjects with MSS or malnutrition risk and sarcopenia are at increased risk of long-term all-cause mortality compared to subjects with either malnutrition/malnutrition risk or sarcopenia. Therefore, MSS may serve as an important prognostic factor in the management of hospitalized older patients. Further studies are warranted to address the association between MSS (with various diagnostic criteria) and other adverse health outcomes, such as disability and frailty, and heath costs in different study populations.

## Electronic supplementary material

Supplementary Tables
