# Supplementary material for: Malnutrition-sarcopenia syndrome predicts mortality in hospitalized older patients
Source: Sci Rep. 2017 Jun 9;7:3171. doi: 10.1038/s41598-017-03388-3 (PMC5466644; doi:10.1038/s41598-017-03388-3)
Supplement: Supplementary file 1 — Supplementary Tables [file 41598_2017_3388_MOESM1_ESM.doc]

**Title page**

**Title:**

Malnutrition-sarcopenia syndrome predicts mortality in hospitalized older patients

**Author list:**

Xiaoyi Hu , MSN; Lei Zhang, MSS; Haozhong Wang, MSS; Qiukui Hao, MD; Birong Dong, MD; Ming Yang, MD

**Supplementary Table S1. Baseline characteristics of participants with or without sarcopenia**

| **Characteristic** | **No sarcopenia**  **(n=370)** | **Sarcopenia**  **(n=83)** | **p** |
| --- | --- | --- | --- |
| Age (years) | 78.4 ± 7.9 | 81.7 ± 6.9 | <0.001 |
| Women | 112 (30.3) | 23 (27.7) | 0.645 |
| Education level | | | |
| Unschooled | 27 (7.3) | 9 (10.8) | 0.237 |
| Primary school | 90 (24.3) | 19 (22.9) |  |
| High school or above | 253 (68.4) | 55 (66.3) |  |
| Current smokers | 41 (11.1) | 13 (15.7) | 0.066 |
| Current alcohol drinkers | 44 (11.9) | 10 (12.2) | 0.445 |
| Physical activity ≥ 30 min/d | 213 (57.7) | 43 (51.8) | 0.326 |
| Comorbidities | | | |
| Hypertension | 200 (54.1) | 49 (59.0) | 0.615 |
| Ischemic heart disease | 112 (30.3) | 20 (24.1) | 0.263 |
| COPD | 105 (28.4) | 33 (39.8) | 0.042 |
| Diabetes | 102 (27.6) | 16 (19.3) | 0.085 |
| Stroke | 22 (5.9) | 6 (7.2) | 0.661 |
| CKD | 49 (13.2) | 8 (9.6) | 0.371 |
| Acute infection | 102 (27.6) | 23 (27.7) | 0.979 |
| Osteoarthritis | 90 (24.3) | 22 (26.5) | 0.677 |
| Tumor of any type | 39 (10.5) | 9 (10.8) | 0.935 |
| GI disease | 70 (18.9) | 19 (22.9) | 0.410 |
| Liver disease | 28 (7.6) | 8 (9.6) | 0.528 |
| Urinary incontinence | 41 (11.1) | 9 (10.8) | 0.950 |
| Chronic pain | 102 (27.6) | 23 (27.7) | 0979 |
| Nutrition status | | | |
| Normal | 205 (55.6) | 14 (16.9) | <0.001 |
| Malnutrition risk | 139 (37.7) | 48 (57.8) |  |
| Malnutrition | 25 (6.8) | 21 (25.3) |  |
| Nutritional supplements | 38 (10.3) | 10 (12.0) | 0.634 |
| BMI (kg/m^2^) | | | |
| Women | 23.8 ± 3.6 | 17.9 ± 2.0 | <0.001 |
| Men | 23.3 ± 3.4 | 18.1 ± 1.6 | <0.001 |
| CC (cm) | | | |
| Women | 32.8 ± 4.1 | 28.0 ± 2.9 | <0.001 |
| Men | 33.3 ± 3.7 | 30.8 ± 3.9 | <0.001 |
| MAC (cm) | | | |
| Women | 27.4 ± 3.6 | 23.7 ± 3.6 | <0.001 |
| Men | 26.8 ± 3.3 | 24.9 ± 3.6 | <0.001 |
| Gait speed (m/s) | | | |
| Women | 0.8 ± 0.3 | 0.5 ± 0.2 | 0.003 |
| Men | 0.8 ± 0.4 | 0.7 ± 0.3 | 0.019 |
| Handgrip strength (kg) | | | |
| Women | 14.7 ± 7.1 | 10.9 ± 7.1 | 0.038 |
| Men | 22.9 ± 8.7 | 18.9 ± 7.2 | 0.002 |
| SMI (kg/m^2^) | | | |
| Women | 5.8 ± 0.7 | 4.4 ± 0.3 | <0.001 |
| Men | 7.4 ± 0.7 | 6.4 ± 0.3 | <0.001 |
| ADL scores | 8.8 ± 3.6 | 9.1 ± 3.6 | 0.370 |
| IADL scores | 10.5 ± 4.8 | 11.0 ± 4.3 | 0.327 |
| GDS-30 scores | 7.5 ± 5.3 | 7.9 ± 5.3 | 0.516 |
| MMSE scores | 24.7 ± 5.6 | 23.5 ± 5.7 | 0.083 |

Data are presented as the number (percent) for the following variables: women, education level, marital status, current smokers, current alcohol drinkers, physical activity, specific comorbidities, and nutritional status. For other variables, the mean ± SD are used.

One-way ANOVA was used for the continuous variables, and the Pearson chi-squared test was used for categorical variables. During testing, p <0.05 was considered statistically significant.

ADL: activities of daily living; BMI: body mass index; CC: calf circumference; CKD: chronic kidney disease; COPD: chronic obstructive pulmonary disease; GDS-30: 30-item Geriatric Depression Scale; GI: gastrointestinal; IADL: instrumental activities of daily living; MAC：mid-arm circumference; MMSE: Mini-Mental Status Examination; SMI: skeletal muscle index

**Supplementary table S2. Nutrition status according to different definitions**

| **Definitions** | **Normal nutrition**  **(n)** | **Malnutrition risk**  **(n)** | **Malnutrition**  **(n)** | **p** |
| --- | --- | --- | --- | --- |
| MNA | 219 | 187 | 47 | 0.965 |
| Modified MNA without BMI | 223 | 184 | 46 |  |

**Supplementary table S3. Factors associated with 3-year mortality according to the Cox Regression Model with a backward stepwise selection (nutrition status was defined by using the modified MNA without BMI)**

|  | **Coefficient** | **SE** | **Wald** | **p** | **HR** | **95% CI for HR** | |
| --- | --- | --- | --- | --- | --- | --- | --- |
|  |  |  |  |  |  | **Lower** | **Upper** |
| Sarcopenia with normal nutrition^$^ | 0.612 | 0.701 | 0.623 | 0.398 | 1.56 | 0.41 | 5.34 |
| Malnutrition risk^$^ without sarcopenia | 0.776 | 0.312 | 9.001 | 0.001 | 2.32 | 1.25 | 4.42 |
| Malnutrition risk^$^ and sarcopenia | 1.554 | 0.587 | 20.113 | 0.000 | 4.14 | 2.18 | 9.21 |
| Malnutrition^$^ without sarcopenia | 0.888 | 0.536 | 4.594 | 0.067 | 2.58 | 0.96 | 7.16 |
| MSS^$^ | 2.341 | 0.617 | 14.873 | 0.000 | 4.56 | 1.91 | 11.21 |
| Age | 1.032 | 0.239 | 9.276 | 0.002 | 1.05 | 1.02 | 1.10 |
| Tumor of any type | 0.896 | 0.359 | 5.282 | 0.031 | 2.01 | 1.08 | 3.98 |

^$^ Nutrition status was defined by using the modified MNA without BMI.

CI: confidence interval; HR: hazards ratio; MSS: malnutrition-sarcopenia syndrome; SE: standard error.

**Supplementary table S4. Sensitivity analyses of multiplicative interaction between each covariate and the “nutrition combined with sarcopenia” status on all-cause mortality**

| **Covariates** | **HR _interaction_** | **95% CI** |
| --- | --- | --- |
| Age | 0.99 | 0.97-1.04 |
| Gender | 1.03 | 0.69-1.32 |
| Education level | 1.10 | 0.93-1.29 |
| Smoking status | 0.92 | 0.82-1.04 |
| Alcohol drinking status | 0.99 | 0.96-1.04 |
| Physical activity status | 0.96 | 0.88-1.13 |
| Hypertension | 1.20 | 0.78-1.44 |
| Ischemic heart disease | 0.89 | 0.56-1.41 |
| COPD | 0.98 | 0.97-1.02 |
| Diabetes | 1.01 | 0.98-10.4 |
| Stroke | 1.11 | 0.90-1.38 |
| CKD | 1.05 | 0.95-1.10 |
| Acute infection | 0.96 | 0.82-1.45 |
| Osteoarthritis | 0.97 | 0.93-1.05 |
| Tumor of any type | 1.12 | 0.86-1.34 |
| GI disease | 0.97 | 0.92-1.04 |
| Liver disease | 0.99 | 0.98-1.01 |
| Urinary incontinence | 0.95 | 0.89-1.11 |
| Chronic pain | 1.01 | 0.96-1.04 |
| ADL scores | 1.19 | 0.87-1.67 |
| IADL scores | 1.04 | 0.96-1.13 |
| GDS-30 scores | 0.99 | 0.97-1.08 |
| MMSE scores | 1.09 | 0.94-1.16 |

ADL: activities of daily living; CI: confidence interval; CKD: chronic kidney disease; COPD: chronic obstructive pulmonary disease; GDS-30: 30-item Geriatric Depression Scale; GI: gastrointestinal; HR: hazard ratio; IADL: instrumental activities of daily living; MMSE: Mini-Mental Status Examination
